# Systematic Review of Gossypol/AT-101 in Cancer Clinical Trials

**DOI:** 10.3390/ph15020144

**Published:** 2022-01-26

**Authors:** Olga Renner, Mascha Mayer, Christian Leischner, Markus Burkard, Alexander Berger, Ulrich M. Lauer, Sascha Venturelli, Stephan C. Bischoff

**Affiliations:** 1 Department of Nutritional Biochemistry, Institute of Nutritional Sciences, University of Hohenheim, 70599 Stuttgart, Germany; olga.renner@uni-hohenheim.de (O.R.); christian.leischner@uni-hohenheim.de (C.L.); markus.burkard@uni-hohenheim.de (M.B.); 2 Institute of Nutritional Medicine and Prevention, University of Hohenheim, 70599 Stuttgart, Germany; mascha.mayer@icloud.com; 3 Department of Internal Medicine VIII, University Hospital Tuebingen, 72076 Tuebingen, Germany; alex.c.berger@gmx.de (A.B.); ulrich.lauer@uni-tuebingen.de (U.M.L.); 4 German Cancer Consortium (DKTK), DKFZ Partner Site, 72076 Tuebingen, Germany; 5 Department of Vegetative and Clinical Physiology, Institute of Physiology, University of Tuebingen, 72074 Tuebingen, Germany

**Keywords:** oral gossypol, AT-101, clinical trial, cancer, oncologic patients

## Abstract

The potential of gossypol and of its R-(−)-enantiomer (R-(−)-gossypol acetic acid, AT-101), has been evaluated for treatment of cancer as an independent agent and in combination with standard chemo-radiation-therapies, respectively. This review assesses the evidence for safety and clinical effectiveness of oral gossypol/AT-101 in treating various types of cancer. The databases PubMed, MEDLINE, Cochrane, and ClinicalTrials.gov were examined. Phase I and II trials as well as single arm and randomized trials were included in this review. Results were screened to determine if they met inclusion criteria and then summarized using a narrative approach. A total of 17 trials involving 759 patients met the inclusion criteria. Overall, orally applied gossypol/AT-101 at low doses (30 mg daily or lower) was determined as well tolerable either as monotherapy or in combination with chemo-radiation. Adverse events should be strictly monitored and were successfully managed by dose-reduction or treating symptoms. There are four randomized trials, two performed in patients with advanced non-small cell lung cancer, one in subjects with head and neck cancer, and one in patients with metastatic castration-resistant prostate cancer. Thereby, standard chemotherapy (either docetaxel (two trials) or docetaxel plus cisplatin or docetaxel plus prednisone) was tested with and without AT-101. Within these trials, a potential benefit was observed in high-risk patients or in some patients with prolongation in progression-free survival or in overall survival. Strikingly, the most recent clinical trial combined low dose AT-101 with docetaxel, fluorouracil, and radiation, achieving complete responses in 11 of 13 patients with gastroesophageal carcinoma (median duration of 12 months) and a median progression-free survival of 52 months. The promising results shown in subsets of patients supports the need of further specification of AT-101 sensitive cancers as well as for the establishment of effective AT-101-based therapy. In addition, the lowest recommended dose of gossypol and its precise toxicity profile need to be confirmed in further studies. Randomized placebo-controlled trials should be performed to validate these data in large cohorts.

## 1. Introduction

**Figure 1:**
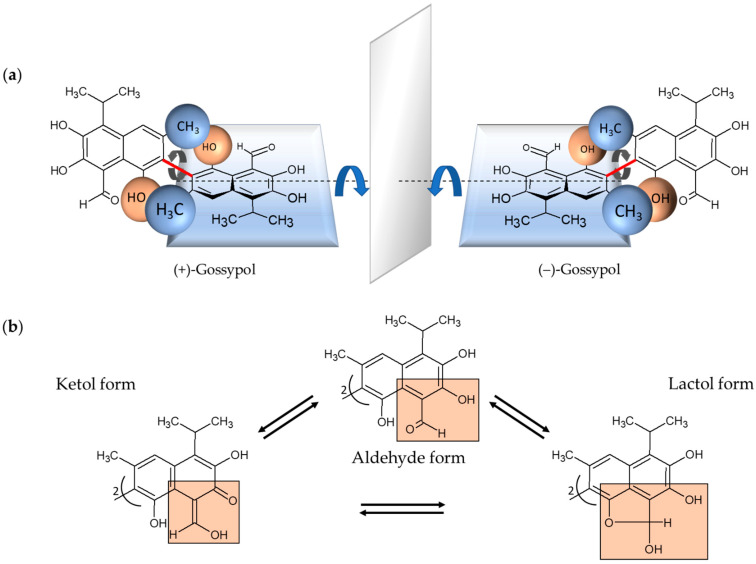
Chemical structural formulas of the enantiomers of gossypol and their tautomeric forms. (**a**) (+)- and (−)-enantiomers of gossypol behave structurally like an image and a mirror image. The rotation around the binaphtyl bond (marked red) is restricted because of the sterically hindering methyl- and hydroxyl-groups of the two naphthalene units (indicated as blue and brown balls for the corresponding methyl- and hydroxyl-groups, respectively). In this special case of axial chirality, the so-called atropisomerism, the formed enantiomers, also known as rotamers, are largely stable. (**b**) Depicted are the tautomeric forms of gossypol, aldehyde, ketol and lactol form, which can be converted into each other. In each case, one of two identical naphthyl residues is shown with the relevant substituents marked in orange boxes.

Gossypol is a complex polyphenolic compound naturally occurring in the glands, leaves, stems, roots, and seeds of cotton plants with the highest concentration in the seeds [1,2]. Gossypol is a strongly colored yellow, crystalline pigment, which is almost insoluble in water and hexane and, in contrast, soluble in acetone, chloroform, ether, and methyl ethyl ketone (butanone). It is also partially soluble in crude vegetable oils. The chemical formula is C_30_H_30_O_8_ and the systematic name is 2,2′-bis (formyl-1,6,7-trihydroxy-5-isopropanyl-3-methylnaphthalene) [2,3]. Due to the restricted rotation of the two naphthyl rings around the interlinking C–C bond, gossypol exhibits atropisomerism (Figure 1) [4].

The two enantiomers, (+) and (−), have been resolved by several groups [5,6,7]. Each enantiomer can exist in solution in three tautomeric forms, the aldehyde-aldehyde, lactol-lactol, and ketol-ketol form, which differ in stability depending on the solvent (Figure 1) [8]. Furthermore, the pharmacokinetic and pharmacodynamic parameters of (+)-, and (−)-enantiomers and their racemic mixture (±) are different [9,10,11,12]. The gossypol (−)-enantiomer—also called AT-101—is degraded more slowly and is therefore the more biologically active form [11]. Consequently, it is also more toxic than (+)-gossypol [1,11]. In the 1950s, gossypol was discovered in China when it was investigated whether cooking with crude cottonseed oil could lead to infertility in men [13]. Subsequently, numerous studies have shown that gossypol not only possesses nutritional properties but is also a potential candidate for biomedical application. Gossypol is reported to show antifertility [14,15], antioxidant [16], antiviral [17,18,19], antiparasitic [19,20,21], and antimicrobial properties [22,23]. Gossypol/AT-101 was used to treat human cancer cells, thereby, anticancer activities were demonstrated in breast cancer [24,25], colon cancer [26], pancreatic cancer [27], and prostate cancer, among others [28]. Gossypol exhibits potent antiproliferative effects in different human carcinoma cell lines [29,30]. Gossypol-induced apoptosis is characterized by cell shrinkage, blebbing, chromatin condensation, and DNA laddering caused by internucleosomal DNA cleavage [4,31], comprising intracellular processes as interaction with Bcl-2 family proteins, induction of the caspase-dependent pathway and mitochondrion-mediated apoptosis, effects on cell cycle and cell signaling pathways, and DNA fragmentation [32,33,34].

AT-101, a natural Bcl-2 homology domain 3 (BH3) mimetic, is a small molecule inhibitor that downregulates anti-apoptotic Bcl-2 and Bcl-2-related proteins in human cancer cells [26,32,35,36,37,38]. Moreover, the levels of other pro-apoptotic (Bcl-XL/Bcl-Xs) proteins could also be upregulated by gossypol and the values of anti-apoptotic factors could be suppressed [32]. Gossypol-induced apoptosis appears to proceed via the caspase-dependent pathway by activation of caspase-3 and caspase-9. [33,39,40,41,42,43]. Another important apoptosis inducing pathway is mitochondrial-initiated endogenous apoptosis. In gossypol-treated cancer cells, alterations on the mitochondrial outer membrane permeabilization (MOMP) cause the release of large amounts of apoptotic markers, such as cytochrome c and apoptosis-inducing factor (AIF), from the mitochondrion into the cytoplasm, as well as mitochondrial membrane depolarization [33,44,45,46]. In addition, gossypol-induced intrinsic apoptosis might occur also as reactive oxygen species (ROS)-independent [33]. Moreover, the suppression of vascular endothelial growth factor (VEGF) stimulating intracellular pro-angiogenic kinases phosphorylation could be inhibited by AT-101 [28,47]. Gossypol initiated Bcl-2 dependent autophagy was described for several malignant cell lines [48,49,50,51,52]. Furthermore, gossypol can affect the cell cycle and cell signaling pathways [39,51,53,54,55,56,57].

Apurinic/apyrimidinic endonuclease 1/redox enhancing factor 1 (APE1/Ref-1, referred to from hereon as APE1) [58,59,60,61,62,63,64] was also shown to be a target of gossypol. Furthermore, gossypol kills cancer cells more effectively when APE1 is overexpressed [58]. Moreover, APE1 overexpression was demonstrated to be associated with cisplatin resistance and the addition of gossypol leads to inhibition of APE1 and enhances the activity of cisplatin in non-small cell lung cancer [61,65,66]. Also, gefitinib sensitivity is enhanced after AT-101 treatment [65,67]. Exhibiting synergistic effects with the alkylating agent cisplatin as well as with the selective inhibitor of epidermal growth factor receptor (EGFR) gefitinib seems to be a promising strategy for further exploration of AT-101-based treatment options in cancer.

In recent years, epigenetic modulation, particularly the modification of DNA-associated histone proteins, has received attention as new targets for cancer therapy. The overexpression of HDAC enzymes, contributing to the silencing of regulatory genes, is often detected in cancer tissues. Therefore, it is of great interest to identify and investigate both synthetic and natural HDAC inhibitors (HDACis) as potential new anticancer drugs [68,69]. Using high-throughput screening of ~1600 non-fermented commonly used nutraceuticals and food-based polyphenols, Mazzio et al. provided evidence of gossypol induced HDACi activity in nuclear HeLa cell lysates [70]. Inhibitory activity against classical HDACs has already been demonstrated for some natural substances, which makes these specific compounds generally very interesting for the investigation of new treatment options of tumor diseases [71,72].

The backbone of cancer therapy includes surgery, chemotherapy, and radiotherapy. Each of these options has distinct limitations due to the presence or establishing of resistances causing treatment failure [73]. The current strategy of combining radiation and/or standard cytotoxic chemotherapeutic agents with phytochemicals, like gossypol, can potentially lead to synergy [74,75,76]. Synergistic effects of gossypol/AT-101 with chemotherapy were demonstrated for conventional chemotherapy in cancer cell lines as well as in animal models [27,35,40,66,77,78,79,80]. Moreover, AT-101 was demonstrated to radiosensitize prostate cancer in vitro and in vivo without augmenting toxicity [81], suggesting that it may improve the outcome of cancer radiotherapy, and proposing gossypol as a potential anticancer regime’s component [57,81,82,83,84,85]. In the meantime, data indicates the benefit of combined, multidrug regimens with inclusion of AT-101 [57,86,87].

In this review, we analyze the translational progress of gossypol/AT-101 treatment from in vivo and animal models into human clinical trials in cancer patients, testing its potential as anti-tumor agent. Thereby, we systemically examine the available data about gossypol/AT-101 application within clinical investigations and focus on clinical outcomes, dose-limiting toxicities, and the relation of gossypol/AT-101 to potential cancer parameters as possible predictable markers of disease status or progression. Finally, we summarize the current data of trials and compare tested regimens against each other, giving an overview of gossypol/AT-101 status in clinical studies.

## 2. Materials and Methods

**Figure 2:**
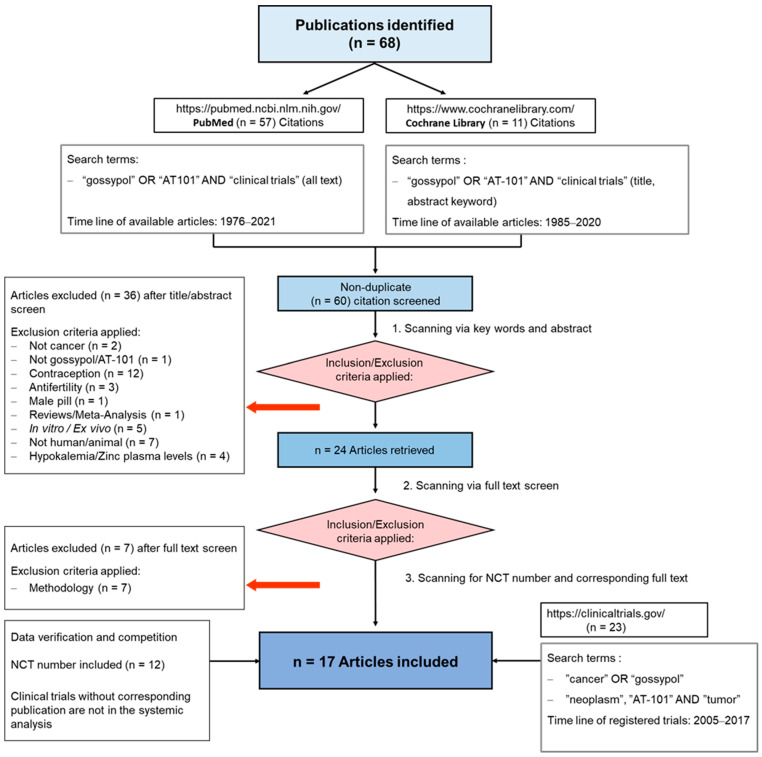
PRISMA Flow Diagram. The diagram illustrates the passage of information through the various phases of this review. It displays the number of studies included and excluded, as well as the criteria for the respective exclusions. The data included in this systematic review were collected from August to September 2021 and reviewed during September 2021.

**Figure 3:**
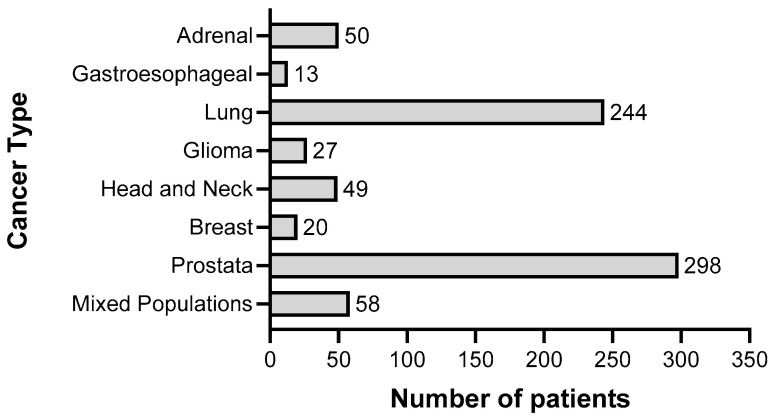
Representation of the total number of patients (n = 759) in relation to cancer type, who were treated with gossypol/AT-101 within clinical trials summarized in this systematic review.

To summarize the results of clinical trials in which cancer patients were treated with gossypol/AT-101, either as a single agent or in combination with standard therapies, a guideline defining the methods of search and analysis of the findings was developed. As applicable for systematic reviews, the key features from the review protocol are entered and maintained as a permanent record under the registration number CRD42021297142 in the PROSPERO database. Patients with a current diagnosis of cancer of any type and stage were determined as an investigational cohort of this review. The application of gossypol/AT-101 alone or in combination with standard cancer therapies was specified as intervention of interest. All human clinical trials comprising uncontrolled or controlled study protocols as well as containing comparisons against no treatment, placebo, or standard of care therapies were accordingly considered during a literature search and are included in the evaluation. Randomized controlled trials were of primary interest, but all study designs were incorporated in the searching procedure. Specifically, the following study outcomes were included in the searching process: Common Terminology Criteria for Adverse Events (CTCAE), adverse events (AE) or serious adverse events (SAE), or other measured parameters like progression-free survival (PFS), overall survival (OS), complete response (CR), partial response (PR), stable disease (SD), progressive disease (PD), objective response rate (ORR), disease control rate (DCR), maximally tolerated dose (MTD), dose-limiting toxicity (DLT), parameters for intact liver function (like serum aspartate aminotransferase (AST), alanine aminotransferase (ALT), and albumin values) as well as Response Evaluation Criteria In Solid Tumors (RECIST). PubMed, MEDLINE, and Cochrane databases were used as platforms, on which an electronic literature search was conducted. PubMed served as an interface for MEDLINE (Figure 2). The exact combination of search terms “gossypol” OR “AT101” AND “clinical trial” were used in PubMed and “cancer”, “gossypol”, and “AT-101” in the Cochrane database. The timing of the literature investigation was August and September 2021, respectively. After all studies with the defined search terms were identified, three authors (OR, MM, and CL) independently inspected the related abstracts, eliminated duplicates, and removed all articles that were not clinical trials or not relevant to the subject matter. Subsequently, a second inspection of the selected literature was done by OR and MM, removing trials that examined tumor models, any animal intervention, antifertility and/or contraceptive, zinc level and gossypol-related hypokalemia, trials with only methodical investigation, and with possible screening parameters related to cancer prognosis but not to gossypol/AT-101 activity. Databases, the U.S. National Library of Medicine, as well as the ClinicalTrials.gov page, and search terms “gossypol”, “AT-101”, “cancer”, and “tumor” were used to find out the available ClinicalTrials.gov Identifier (NCT number) and to supplement or monitor further study details. After the second screening, the remaining studies were summarized using a narrative approach. OR and MM created independent excel spreadsheets and compared these with each other and completed them in case of lacking information, generating a master file. OR, MM, CL, and MB were responsible for the third comprehensive proof of all entered data in the final master sheet as a quality assessment. A total of 17 articles that included 759 patients evaluated efficacy and toxicity of gossypol/AT-101 (Table 1 and Table 2). All of these trials investigated orally dosed gossypol/AT-101. The median sample size of these studies was 45 subjects (ranging from 13–220) and the orally given gossypol/AT-101 dose ranged from 10 mg daily [57] to 40 mg every 12 h [88], with a maximum daily dose of 180 mg [89]. These clinical trials were conducted in the time period from 1992–2021, examining the role of gossypol/AT-101 in a total of eight tumor entities: lung cancer (n = 5), advanced human cancer (n = 2), prostate cancer (n = 3), metastatic adrenal cancer (n = 2), head and neck cell carcinoma (n = 2), breast cancer (n = 1), glial tumors (n = 1), and gastroesophageal carcinoma (n = 1) (Figure 3).

## 3. Results

### 3.1. Trials Determining Therapeutic Effect and Toxicity of Single-Agent Gossypol/AT-101

Out of the 17 clinical trials included in the total analysis, in seven studies with a total of 168 individuals, patients were treated with gossypol/AT-101 as mono-therapeutic agent to evaluate possible dose-limiting toxicities and its clinical activity as an anti-tumor substance. In the study design of these trials, gossypol/AT-101 was regarded either as an inhibitor of DNA replication or repair [54,89] as a selective inhibitor of intermediary metabolism [93], as a potent inhibitor of Bcl-2 family apoptosis related proteins (BH3 mimetic) [90,91,92], or as DNA silencer [54]. Within these clinical trials, patients either with advanced or unresectable, or metastatic, or refractory tumors of different entities were included to validate the benefits of gossypol’s therapeutic activity.

Firstly, described by Stein at al., gossypol was tested in a total of 34 patients with histologically proven advanced malignancies who had failed to respond to conventional systemic treatment or for whom no effective systemic treatment was available [89]. Thereof, most patients suffered from cancer of the digestive apparatus (n = 14), lung (n = 10), or breast (n = 7). Based on Chinese contraceptive trials at a dose of 20 mg/day [103], racemic gossypol acetic acid was administered to participants by weekly escalating doses of gossypol ranging from 30 to 180 mg. In the second part, subjects were treated with repeated doses, which were initially given twice weekly, then daily, and finally, 30 mg twice daily. The dose of 30 mg twice daily was determined as safe for the administration in patients. Toxic side effects included emesis, diarrhea as well as lethargy and seemed to be dose-related. Serum gossypol levels were measured at approximately 24 h after dosing and were generally rather lower than those that have been used in growth-inhibitory studies in tissue-culture models. AT-101, the R-(−)-enantiomer, was determined to have a higher therapeutic index than the R-(+)-enantiomer and was suggested as an agent for further clinical trials. As racemic gossypol acetic acid failed to show a clinical activity in the total cohort, it was speculated that gossypol is able to develop more activity in patients with more favorable disease status.

Based on this preliminary clinical study, two clinical trials evaluated the therapeutic effect of AT-101 in adrenal cancer. Flack et al. started with a dose-escalating regimen, beginning with 20 mg/day and increasing every two days to 30–70 mg/day in divided doses [94]. Thereby, the maximum tolerated gossypol dose was determined to be 0.8 mg/kg per day (50–60 mg/day), and it correlated only roughly with the prescribed dose but did not significantly decrease steroid excretion measured in the urine. Of the 18 patients who had measurable gossypol levels, no patient had to permanently discontinue gossypol due to its side effects. However, the most common side effects were transient transaminitis (93%), xerostomia (93%), followed by dry skin (71%), fatigue (64%), intermittent nausea (36%), vomiting (21%), transient ileus (21%), and minor hair thinning (14%). Thus, in this investigated cohort of metastatic adrenal carcinoma, the observed partial tumor response rate was of 17% over the period of several months or one year. These partial responses were seen at doses 0.6–0.8 mg/kg per day (40–60 mg/day), however were quite variable, ranging from 83–547 ng/dL. Therefore, the clinical activity of gossypol was suspected and this treatment regimen was suggested for daily usage when other therapies have failed.

In contrast, the more recent investigation of Xie et al. tested 20 mg/day of oral AT-101 for 21 days out of 28-day cycles in patients with advanced adrenal cortical carcinoma [90]. Seven percent of the AT-101 treated patients experienced grade 4 toxicity (e.g., increase in cardiac troponin levels and hypokalemia). In four patients, the dose was reduced in four treatment cycles (5%) due to grade 3 nausea and vomiting, grade 3 hypokalemia, elevated AST/ALT, and/or fatigue. After conduction of the interim analysis subsequently to the finalization of the first two study stages, none of the first 21 patients achieved PR (as defined per RECIST criteria). Therefore, this protocol was considered as not effective and the trial was prematurely stopped. Also, in patients with chemotherapy-sensitive recurrent extensive-stage small cell lung cancer, the failure to meet the primary endpoint at the interim analysis by testing of 20 mg/day orally AT-101, for 21 out of 28 days per cycle, lead to a premature discontinuation of the study [91]. No grade 4 toxicities were observed within this clinical investigation. Besides hematological and non-hematological grade 3 and 4 AEs, no ORs were observed and only three patients (21%) achieved SD after two cycles, but subsequently progressed on treatment. The median time until progression was 1.7 months, and median OS was 8.5 months.

Furthermore, three trials examined the therapeutic benefit of gossypol/AT-101 in recurrent adult malignant gliomas in breast and in castrate-resistant prostate cancer [54,92,93]. Notably, there were a further three different tumor entities and three different dose regimens tested (first: 10 mg twice daily, as continuous procedure; second: daily doses between 30–50 mg; third: daily at 20–30 mg for 21 of 28 days as one cycle). In all of these trials, differently and heavily pre-treated as well as poor-prognosis subjects were included. Mild toxicities were observed when treating with 10 mg daily [93]. When patients were treated with 30, 40, or 50 mg gossypol, grade 1–2 toxicities included nausea (30%), fatigue (15%), emesis (15%), altered taste sensation (15%), and diarrhea in (10%) of patients. Receiving 50 mg/day, two patients experienced dermatologic grade 3 DLT [54]. In the phase I trial, castrate-resistant prostate cancer patients started with 30 mg daily on 21 days of a 28 day’s cycle and showed increased gastrointestinal toxicity [92]. As a result, the phase II starting dose was chosen to be 30 mg. Due to the frequent occurrence of the AEs (any grade), diarrhea (43.5%), fatigue (34.8%), nausea (21.7%), anorexia (21.7%), small intestinal obstruction (21.7%), and high incidence of grade 3 small intestinal obstruction (21.7%), a dose reduction to 20 mg was authorized for all patients [92]. At the 30 mg twice daily dose, two of three patients developed grade 4 AST/ALT elevation associated with nausea and vomiting after one week. Grade 4 hypokalemia and grade 3 nausea were observed when 40 mg was given daily, so the dose was reduced to 30 mg. None of these three trials showed a significant benefit after gossypol treatment was achieved. Nevertheless, the trial’s conductors defined an MTD as 40 mg/day [54] and they were able to induce a partial response or temporary stabilization of disease in the same cancer patients, respectively.

In a total of seven clinical trials that evaluated gossypol monotherapy in cancer patients, an orally given tablet was used as a drug administrating method., with the exception of Stein et al. [89], where racemic gossypol acid was purified to standard pharmacological levels and packaged in solid form into gelatin capsules in 30 mg doses. Ten mg of racemic gossypol acetic acid, compressed to or incorporated in a tablet (obtained from the Chinese Academy of Medical Sciences) (Beijing, China), was defined as the initial testing unit [54,93,94]. As recommended by Stein [89], four clinical trials administered [90,91,92,98], explicitly AT-101, the levorotatory enantiomer of gossypol. In this regard, Xie et al. [90] and Baggstrom et al. [91] referred to National Cancer Institute as the supplier of the test compound AT-101 (NSC# 726190). In summary, there are two trials that were prematurely stopped due to the failure to achieve the defined primary endpoints [90,91]. Although the other five did not observe a significant difference, they suggested a further investigation of gossypol as an antitumor agent [89,94], probably in combination with other neoplastic agents [54,92].

### 3.2. Trials Evaluating Efficacy and Safety of Gossypol/AT-101 in Combination with Standard Chemo- and Radiation Therapies in Cancer Patients

Ten clinical trials, from 2010–2021, investigated the safety and efficacy of AT-101 in combination with conventional anti-tumor therapies. Altogether, three times (n = 591 subjects with a median n = 65) as many patients participated in these studies. In three trials, AT-101 was tested combined with one standard chemotherapeutic (1× topotecan, 2× docetaxel) [97,101,102]. Double standard treatment in combination with AT-101 was investigated in four studies [88,96,99,100]. Notably, one cisplatin and radiation regimen was established [57]. The recent one, analyzed radiation combined with docetaxel, fluorouracil, and AT-101, was administered as a four-component regimen [95].

Four trials examined the activity of AT-101 in lung cancer. For the eldest one, a phase I/II study was conducted, combining AT-101 with topotecan in relapsed and refractory small cell lung cancer [102]. In parallel, as described above, AT-101 monotherapy was studied in a phase II trial in refractory small cell lung cancer, which was performed concurrently by Baggstrom et al. [91]. In the current open-labelled multicenter phase I/II study, during the phase I stage, an initial dose of 1.25 mg/m^2^ topotecan (intravenously over 30 min), in combination with 40 mg of oral AT-101 was administrated for five consecutive days (1–5) of a 21 days cycle [102]. Higher AT-101 concentrations were not tested due to reported dose-limiting AT-101 associated toxicities [54,92]. Due to the occurrence of AEs/intolerances, one dose reduction of AT-101 to 30 mg/day was permitted by the study protocol. Assessment of the therapeutic response involved RECIST criteria. In at least 10% of the study participants, AEs were detected. The most common toxicities had a hematological background, as would be expected in a combination of topotecan and AT-101. Gastrointestinal side effects were also usual, although most of these were grade 1 and 2. At least one therapeutic response had to be observed in the first stage to continue to stage 2 and at least six treatment responses had to occur in both stages to justify further investigation in future studies. In the sensitive relapsed cohort (n = 18), there were zero CRs, three partial responses PRs, ten SDs, and four PDs. In the refractory cohort (n = 12), there were zero CR/PR, five SD, and five PD. As this study did not meet its pre-specified efficacy criteria, the continuation of enrolment was stopped after the first stage of the two-stage phase II design. However, the authors of the article finished their article by pointing out that, despite the lack of significance, two patients benefited from the treatment with long progression-free survival and prolonged responses.

Schelman et al. conducted a phase I study assessing the therapeutic role of AT-101 in combination with cisplatin and etoposide [99]. During dose escalation, increasing doses of AT-101 (30–40 mg twice daily) were administered orally on days one to three along with cisplatin (60 mg/m^2^) on day one and etoposide (120 mg/m^2^) on days one to three of a 21-day cycle in 20 patients with advanced solid malignancy, refractory to standard therapy or for which no curative standard therapy was available. Eight participants from dose escalation cohort had small cell lung cancer. Due to early DLT of febrile neutropenia, the protocol was amended to include the administration of filgrastim of all subsequent cycles. At the second stage, the preliminary activity of a triple therapy consisting of AT-101, cisplatin, and etoposide was assessed in an expanded cohort of patients with extensive-stage small cell lung cancer. Using the regimen of AT-101 with 40 mg twice daily at the first three days of the 21-day cycle, in combination with cisplatin and etoposide, and supplementation of filgrastim, antitumor activity was observed in a variety of cancers including patients with advanced solid tumors as well as study subjects with extensive-stage small cell lung cancer. Grade 3 and 4 treatment-related toxicities such as diarrhea, increased AST, neutropenia, hypophosphatemia, hyponatremia, myocardial infarction, and pulmonary embolism were detected. Due to the high rate of thrombotic complications that occur in the setting of advanced cancer, attribution of these events to AT-101 is difficult. With the used AT-101 concentration, no significant interactions were observed with cisplatin and etoposide in cycle 2.

In addition to small cell lung cancer, there are two further double-blind, placebo-controlled, randomized phase II studies evaluating pharmacological activity of gossypol/AT-101 in non-small cell lung cancer [96,101]. Ready at al. used a regimen of AT-101 (40 mg twice daily for three days) or placebo in combination with docetaxel (75 mg/m^2^ on day 1) every 21 days [101]. The most frequent AEs were fatigue (18%), anemia (18%), and dyspnea (18%). No statistically significant differences in SAEs were observed in the AT-101 and the placebo group. In contrast to clinical trials with continuous daily AT-101 application, no development of small bowel obstruction was reported. In the study of Wang et al., 31 patients in the experimental group received 75 mg/m^2^ docetaxel and 75 mg/m^2^ cisplatin on the first day combined with 20 mg gossypol once daily from days one to 14 of a 21 days cycle [96]. The control group received placebo with the same docetaxel and cisplatin regimen. There were no treatment-related deaths or discontinuation of therapy protocols due to toxicity. Most patients developed only mild AEs (grade 1 and 2) and only one patient experienced grade 3 anemia in the gossypol group. No significant increase in toxicity in the gossypol group compared with the placebo group was noted. In both trials, the experimental group had a better but not significant outcome regarding RECIST criteria and regimens of gossypol combined with docetaxel and cisplatin or of AT-101 and docetaxel were well tolerated. However, the compatibility of these two trials is limited not only due to the second chemotherapeutic cisplatin but also due to the origin and amount of the tested agent. In contrast to Wang et al., where gossypol acetate tablets of 20 mg were applied once daily [96], a total of 80 mg, divided in two portions of R-(−)-enantiomer/AT-101, were tested by Ready et al. [101].

The largest clinical trial that investigated AT-101 included 220 study participants and was a double-blind, placebo-controlled phase II study with two-arms and 1:1 randomization [100]. Male participants with metastatic castration-resistant prostate cancer were treated with docetaxel (75 mg/m^2^ day 1) and prednisone 5 mg orally twice daily every 21 days in addition to 40 mg AT-101 or placebo twice daily on days one to three of a 21 days cycle. Disease progression was defined in accordance with the Prostate Cancer Clinical Trials Working Group (PCWG)-2 recommendations according to RECIST, and worsening according to Eastern Cooperative Oncology Group (ECOG) performance status. The incidence of distinct grade 3/4 AEs increased in the triple regimen compared with placebo including cardiac AEs (5% vs. 2%), lymphopenia (23% vs. 16%), neutropenia (47% vs. 40%), ileus (2% vs. 0%), and pulmonary embolism (6% vs. 2%). The incidence of grade 1/2 peripheral neuropathy was 24% vs. 13%. Neutropenic fevers were uncommon and occurred in 2.7% of the patients in both arms. The median number of cycles was eight in the triple regimen arm and nine in the placebo arm. In the triple regimen, a higher proportion of patients that discontinued therapy (27.3% vs. 16.4%) or underwent dose reductions (21.8% vs. 13.6%) due to AEs was found. Regarding the primary end point of the study, defined as increased OS, and secondary end points defined as median of PFS, reduction of prostate-specific antigen (PSA) levels, measurable DCR, ORRs as well as pain response rates, no significant differences were noted. A potential benefit was observed in the high-risk patient group, with OS of median 19 vs. 14 months as well as for other secondary end points.

The third clinical trial of prostate cancer was performed in a cohort of patients with castration sensitive metastatic prostate cancer [98]. In contrast to previous prostate trials [92,100], where chemotherapy, naive castrate-resistant prostate men were included, only patients with newly diagnosed cancer and prior local therapy with radiation or surgery were allowed in this study. Herewith, clinical activity of AT-101 in combination with an androgen deprivation therapy (ADT) for metastatic prostate cancer was examined. Subjects were permitted to initiate anti-tumor therapy with standard of care ADT up to six weeks prior to beginning treatment with AT-101. ADT was prescribed according to physician decision as a commercially available luteinizing hormone-releasing hormone (LHRH) agonist. Daily oral bicalutamide 50 mg was required during the first month of LHRH agonist therapy. AT-101 was administered orally 20 mg/day on days one to 21 of a 28-day’s cycle. Patients received up to eight cycles of AT-101 treatment. PSA level was defined as the primary endpoint to evaluate treatment response, but the patients were as well monitored by objective disease status. Moreover, treatment was discontinued in 35% (19/55) of patients due to AEs. Twelve patients (22%) experienced SAEs and eleven patients (13%) experienced SAEs considered to be related to AT-101 containing therapy. Although the combination of AT-101 and ADT failed to reach the pre-specified level of activity (only 31% of study participants achieved undetectable PSA levels), further investigation of AT-101 in combination with ADT was recommended.

Previously, Stein and colleagues tested gossypol/AT-101 as single agent in a cohort with advanced solid tumors or in men with newly diagnosed castration-sensitive metastatic prostate cancer [89,98]. In a third study, the therapeutic activity of AT-101, as a triple therapy, was evaluated [88]. Thereby, AT-101 was administered constantly with 40 mg every 12 h on days one to three of each cycle combined with varying dose levels of paclitaxel and carboplatin on a 21-day cycle. Due to hematologic related AEs, twelve (50%) patients received filgrastim or pegfilgrastim therapy, and two patients (8%) were treated with erythropoietin, filgrastim, or pegfilgrastim. In the pharmacokinetic studies, plasma gossypol levels remained to continually rising up to 10 h, indicating abnormal absorption and elimination of AT-101. Also, paclitaxel pharmacokinetics were not altered by the oral administration of AT-101. In addition, the pharmacodynamic analysis did not reveal any statistically significant decreases of Bcl-2 and Caspase 3 protein levels or increased apoptotic activities induced by AT-101 as it was demonstrated in preclinical studies [104]. While disease control was achieved only in a subset of the study cohort, especially in docetaxel refractory prostate cancer patients, the clinical benefit seen from this and other phase I studies [97,102] was regarded as AT-101 independent and defined as modest.

Docetaxel (75 mg/m^2^) in combination with 40 mg AT-101 given twice daily was already tested for clinical efficacy in patients with advanced or metastatic non-small cell lung cancer [101], either as a compound of a triple regimen, containing AT-101, docetaxel (75 mg/m^2^) IV and prednisone (5 mg), or docetaxel (75 mg/m^2^), and cisplatin (75 mg/m^2^) [96,100]. In an open label randomized phase II trial, patients with locally advanced or metastatic head and neck cancer were treated with docetaxel (75 mg/m^2^), docetaxel (75 mg/m^2^) plus pulse dose AT-101 (40 mg twice daily on days 1–3 of 21-day cycle), and docetaxel (75 mg/m^2^) plus metronomic dose AT-101 (20 mg daily on days 1–14 of 21-day cycle) [97]. As the primary endpoint, the clinical benefit (comprising CR, PR, or SD established by RECIST) associated with an AT-101 regimen was defined. Secondary objectives included survival, toxicity, and quality of life. Treatment with AT-101 containing regimens was tolerated relatively well with only 6% (two patients) discontinuing treatment due to toxicity. Twelve patients required dose modifications (23%), mostly due to hematological toxicities. Hematological toxicities were the most common treatment related toxicities, of which eleven episodes of grade 3/4 lymphopenia and five episodes of grade 3/4 anemia were noted. In line with previously described studies in the current cohort, the addition of AT-101 to docetaxel did not demonstrate significant evidence of efficacy.

Radiation is a fundamental treatment option to reduce inoperable advanced malignancies. Using two head and neck squamous cell carcinoma lines, Zerp et al. showed that AT-101 enhances radiation-induced apoptosis and demonstrates a radio-sensitization [57]. In addition to these in vitro analysis, 13 patients with locally advanced inoperable head and neck squamous cell carcinoma of the oral cavity, oropharynx, or hypopharynx and no prior radiotherapy to the head and neck region or prior cisplatin-based chemotherapeutic treatment were enrolled. Therefore, 70 Gy were delivered in 35 fractions over seven weeks, concurrently with three-weekly 100 mg/m^2^ cisplatin and combined with dose-escalating oral administration of AT-101 (10 or 20 mg) for two weeks daily every three weeks. Data received from pharmacokinetic analysis at daily doses of 10–20 mg showed that plasma levels peaked around 2 h after intake, suggesting slow absorption and maximum plasma concentrations were in the micromolar range, corresponding to those that induced radio-sensitization in prior in vitro investigations [81,82,85,86]. Based on these findings, the trial conductors encouraged the further evaluation of AT-101 administration in combination with a standard chemo-radiation regimen [57].

In the more recent pilot clinical trial, 13 patients with locally advanced esophageal or gastro-esophageal junction cancer, who were not deemed suitable for surgery, received AT-101 concurrently to chemo-radiation [95]. Patients with metastatic cancer were excluded. Therefore, chemo-radiation therapy contained docetaxel (20 mg/m^2^ as bolus once a week × 5), fluorouracil (225–300 mg/m^2^ as low-dose continuous infusion daily from Monday through Friday × 5) and a radiation dose of 50.4 Gy in 28 fractions. AT-101 (a total of 10 or 20 mg daily) was taken orally on five consecutive days of each week of chemo-radiation. DLTs were not observed for 10 mg AT-101. The most common AEs were related to the gastrointestinal tract, including vomiting, anorexia, and odynophagia. A total of nine serious AEs were encountered irrespective to AT-101. No AEs required dose reduction and no DLT was experienced or established. The encouraging study outcome including achievement of cCR in eleven patients and downregulation of biomarkers in the post-treatment tissues confirmed the previously described mechanism (suppression of yes-associated protein 1/SRY-box transcription factor 9 (YAP1/SOX9) signaling axis) in vitro as well as in a mouse model and suggested AT-101-related downregulation of cancer stem cells. The median duration of clinical complete response was 12 months (range 3–59 months) and their survival was much longer as expected. With the median PFS time of 52 weeks, recurrences were noted in ten of 13 patients. None of the clinical variables correlated with OS or PFS.

Taken together, a total of ten clinical trials explored the safety, tolerability, and therapeutic activity of AT-101 in combination with other established anti-tumor therapies. First, besides the differences in quantity, frequency, and duration of administrated AT-101 dose and irrespective of therapy agent as well as of tumor entity, eight trials reported only a modest clinical efficacy. Second, with exception of Stein et al. [98] and Swiecicki et al. [97], AT-101 could be safely combined with conventional applied anti-tumor regimes and was well tolerated by patients, independently of anti-tumor agent. Third, based on the identification of Zerp et al. [57], AT-101 seems to be a competent enhancer of radiation-induced apoptosis. Fourth, and most intriguing, synergizing regimes of radiation, docetaxel, fluorouracil, and 20 mg AT-101 daily demonstrated clinical CRs in eleven (of 13) patients and prolongation of their OS, reducing the expression of cancer stem cells genes in specimens of treated subjects [95].

### 3.3. Investigations Not Published as an Original Manuscript/Work on PubMed, MEDLINE, and Cochrane Databases but Containing Results of Clinical Investigations

In addition to literature research in the PubMed, MEDLINE, and Cochrane databases, the ClinicalTrials.gov, a resource provided by the U.S. National Library of Medicine, was screened for registered studies, testing gossypol/AT-101 as a potential anticancer agent. Therefore, the search terms “gossypol”/“AT-101”, and “cancer” or “tumor” were entered to identify the relevant investigations. From 2005–2015, a total of 23 clinical trials were included in the database. In some articles ClinicalTrials.gov identifiers/NCT numbers were missing and other relevant information on dosing regimen or details on toxicities were included in the narrative description or also for comparison of the studies of the current systematic review. As the focus of the review was on outcomes, studies with “has results” status were examined in detail. After determining searching criteria, seven of 23 trials were defined to own the results and four from seven were already described and published as an original work. The remaining three studies were explicitly proven for outcome.

In the open-label, monocentric phase II trial (NCT00988169), the efficacy of the combination with erlotinib and AT-101 was investigated in patients with advanced non-small cell lung cancer who were treatment-naive, and had epidermal growth factor receptor activating mutations. In the first stage of the study, patients were scheduled to receive oral erlotinib 100 mg daily and pulsed doses of oral AT-101 given 40 mg twice daily on days one to three of a 21-day cycle. If the initial combination of erlotinib and AT-101 was well tolerated, a dose increase of erlotinib to 150 mg daily was planned for the second cycle. Patients were allowed to receive treatment until they refused further therapy, developed progressive disease, or developed unacceptable toxicity. Based on data posted on the ClinicalTrials.gov home page, six patients started with the treatment, and one subject subsequently withdrew. Five patients finished the treatment; one minor response, three stable diseases, and one partial response were noted. Regarding toxicities, one cardiac SAE (hypertension) and three different types of AEs were noted: blood and lymphatic system disorders (abnormalities in ALT/AST, neutrophils/granulocytes), gastrointestinal disorders (diarrhea), and general disorders (fatigue, asthenia, lethargy, malaise, and nausea). No further information is accessible from this clinical trial.

In the phase I/II trial (NCT01003769), for the patients with B-cell chronic lymphocytic leukemia, the optimal dose of lenalidomide should be evaluated, when combining it together with AT-101. Therefore, patients received 5 mg lenalidomide orally once on days 1–21 at dose level 1. Starting from course 2, patients were planned to also receive 40 mg AT-101 orally twice a day on days one to three and to repeat this treatment every 28 days for up to 11 courses (49–56 days for course 12 or last course of treatment) as long as they did not show DP or signs of unacceptable toxicity. In a total of five participants, no all-cause mortality was noted, however two SAEs and several AEs of eight different origins were noted for each patient. Blood and lymphatic system disorders were particularly common; ALT/AST and bilirubin levels were increased, and neutrophil and platelet levels decreased. This trial was terminated early with an insufficient number of patients to analyze the defined endpoint.

Lastly, in a single group, an open label phase II study, AT-101 was given to patients with recurrent glioblastoma multiforme. A total of 56 participants were enrolled and received oral gossypol once daily on days 1–21. The planned regimen should have been repeated every 28 days in the absence of disease progression or unacceptable toxicity. From a total of 56 participants analyzed, five were not evaluable, not one subject established complete response, one had a partial response, 15 had stable disease and 35 patients progressed. Six SAEs (cardiac, ileus, fatigue, hypophosphatemia, and seizure) were noted and 26 AEs were apparent (gastrointestinal disorders, fatigue, anorexia, peripheral sensory neuropathy, as well as skin and subcutaneous tissue disorders). As not enough tumor tissue was reserved and survival outcomes did not allow the analysis of the pharmacokinetics, data was not displayed by the study investigators.

One study (NCT00286780) with a corresponding abstract was identified. In contrast to the other studies, this article was the first that provided evidence for the administration of AT-101 in non-solid tumors. Based on previously established data from in vivo models, AT-101 was shown to enhance the cytotoxicity of rituximab in chronic lymphocytic leukemia cells in vitro [105]. In another phase II, open label study the combination of AT-101 with rituximab was evaluated in patients with relapsed or refractory chronic lymphocytic leukemia via two dose regimens [106]. In the first part of the study, twelve patients received 30 mg AT-101 on days one to three for four weeks and up to 12 weeks, and rituximab, 375 mg/m^2^ for 12 doses (total dose = 4500 mg/m^2^) on days one, three, five, eight, 15, 22, 29, 31, 33, 40, 57, 59, and 61. In a second study group (n = 6), “pulse” AT-101 was administered (80 mg/day on days one to three and 15–17 of each 28-day cycle) in combination with weekly rituximab (375 mg/m^2^). In the “30 mg AT-101” study group, gastrointestinal toxicity was the most frequent AE, even ileus grade 3–4 was detected in 2/12 participants. Interestingly, only NCI-CTCAE grade 1/2 toxicity was noted in the “pulse” AT-101 group. After 80 mg of AT-101, plasma concentrations of up to 6.6 μM have been observed compared with concentrations of approximately 0.8–1.8 μM after a 30 mg dose in the daily dose cohort. In the “pulse” AT-101 cohort, PR were observed in three patients while the other three were still receiving treatment. Five out of 12 patients had PR in the “30 mg AT-101” continuous administration group. When comparing the daily dosing, intermittent administration of AT-101 with a “pulse” dose regimen appeared to be associated with an increased pro-apoptotic effect in vivo and with higher plasma concentrations, as well as with reduced toxicity.

## 4. Summary

Clinical trials that have examined the use of oral administrated gossypol/AT-101 in cancer patients suggest its potential to exhibit anti-tumor activity only in a subset of patients [88,95,96,99,107]. Gossypol was used either as 30 mg racemic gossypol acetic acid compressed into tablets (Palmer Research Laboratories) [89], or as 10 mg racemic gossypol acetic acid, compressed to or incorporated in tablets (obtained from the Chinese Academy of Medical Sciences) [54,93,94]. AT-101 (NSC# 726190) was supplied by the National Cancer Institute Cancer Therapy Evaluation Program as tablets containing 10 mg of the drug [88,90,91,96], or as 10 mg immediate release tablets (Ascenta Therapeutics, Inc., Limousin, France) taken at the same time each day [92,100], or as 20 mg gossypol acetate tablets produced by Xi’an Northern Pharmaceutical Co., Ltd., Xi’an, China) [96]. If it was mentioned in the treatment plan, gossypol/AT-101 formulation was administered at least 1 h prior to or 1 h after meals [92,93,99,100] and prior to i.v. administration [88]. The solubility of poorly soluble active agents depends, among other things, on the particle size and the particle wettability. Since gossypol is almost insoluble in water, one possibility to increase the solubility of the gossypol preparation is micronization, even though this information is not included in the studies reviewed. The phrase “immediate release tablet” [92,100] might suggest this and would be consistent with the data from Yang et al. [108]. Regarding registered clinical trials available on the ClinicalTrials.gov home page, there are no upcoming or active interventional trials utilizing gossypol/AT-101 in cancer patients.

**Figure 4:**
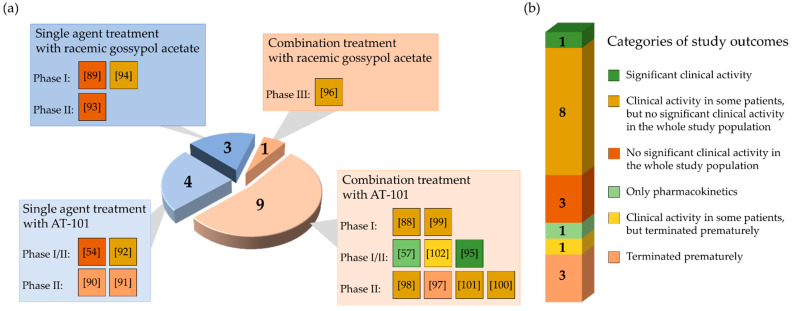
Graphical representation of the results of reviewed studies in cancer clinical trials analyzing the administration of gossypol/AT-101 as a single agent or in combination with standard anticancer treatments. The pie chart is stratified according to study design (**a**) and outcome (**b**). (**a**) The diagram illustrates the total number of conducted clinical trials as a circle. The numbers in the four sections correspond to the number of studies performed in each case. The light blue sections represent the proportion of studies performed with gossypol/AT-101 as single agent, light orange sections represent the proportion of combined therapies (gossypol/AT-101 and standard anticancer protocols). Each section is further subdivided by study phase and contains colored boxes with the corresponding references in brackets. Each color represents one of the study outcomes defined in (**b**). (**b**) The bar divides the study outcomes into six categories. Each of the six different categories contains the number of studies performed and is color-coded according to the study outcome.

From 17 clinical studies investigating the therapeutic potential of gossypol/AT-101 in oncologic patients, there is one trial demonstrating a significant benefit regarding RR or SD, or prolongation of survival [95] (Figure 4).

After a long period of testing of gossypol/AT-101 against different tumor entities as monotherapy or in combination with other anti-tumor drugs, Song et al. recently demonstrated an encouraging success regarding the treatment of patients with gastroesophageal carcinoma. In contrast to other investigations, where the benefits of the treatment were observed either in some subjects or in a special sub-group of patients, there are at least three major differences in this new study. For the first time, the researchers focused on cancer stem cells in addition to anti-apoptotic pathways, which often cause therapy residence. Second, besides the standard chemotherapeutic regimes of intravenously applied docetaxel and fluorouracil, study participants also received a radiation dose of 50.4 Gy in 28 fractions and oral AT-101 at 10/20 mg, daily. Third, using an in vitro and in vivo xenograft model and a pilot clinical phase I trial, the mechanism of action was demonstrated. Thereby, AT-101 appears to target cancer stem cells by abrogating YAP1/SOX9/β-catenin signaling in addition to suppress anti-apoptotic signaling even when Bcl-2 is downregulated. The observed overexpression of YAP1 and SOX9 in untreated specimens and downregulation of YAP1 and SOX9 in patient specimens after treatment, in vitro as well as in vivo, suggest the triple combination of AT-101 with chemotherapy and radiation as worthy of further study.

In total, four clinical trials were terminated because the pre-specified primary endpoints were not met at the time of interim analysis [90,91,97,102] (Figure 4). Even though the tested regimen did not show significant success, the further investigations on AT-101 as an antitumor agent were generally considered a promising strategy. To test gossypol/AT-101 as a mono-therapeutic in cancer patient, seven clinical studies were performed and ten investigated the clinical activity of AT-101 combing standard anti-tumor regimens. Four phase I, five phase I/II, seven phase II, and one phase III clinical trials were conducted. From there, a randomized, double-blind, placebo-controlled design was applied in four studies [96,97,100,101].

### 4.1. Bioavailability, Digestion, Transporters, and Liver Toxicity

From animal studies, it is known that gossypol feeding causes a reduction in the uptake of glucose, alanine, leucine, and calcium, affecting the activities of sucrase, lactase, maltase, and alkaline phosphatase as well as a decrease in the enzyme velocity [109]. Gossypol decreases the Na^+^-dependent active glucose uptake [110] and also alters the ion transport [111]. The half-life of gossypol in the elimination phase following oral administration is relatively long, suggesting that gossypol exerts high plasma and tissue protein binding that prevents it from being eliminated from the bloodstream [112]. The low oral bioavailability of gossypol may also be explained by its relatively low trans-membrane permeability across the intestinal epithelium, relative instability under the weakly basic conditions found in the small intestine, and/or hepatic first-pass metabolism when administered orally [112]. Few studies have examined the exact extent of the oral bioavailability of gossypol and its stereoisomers, and most of these studies have been conducted in animals ((±)-gossypol in dogs: 30.9 ± 16.2%, in mice: 12.2–17.6%) [11,112]. Gossypol nanosuspensions can be absorbed in whole intestinal sections and are able to permeate across the intestine without being affected by p-glycoprotein (P-gp) efflux [108].

High uptake in reticuloendothelial system organs was also described [108]. The pharmacokinetic analysis indicates that gossypol undergoes extensive extravascular distribution, is thereby cleared from the plasma compartment and may react with basic amino acids to bind to target proteins [112,113]. Kinetic findings indicate that gossypol does not compete with ATP, Mg^2+^, Na^+^, and K^+^, but inhibits the enzyme activity of (Na^+^ and K^+^) ATPase, elucidating the hemolysis in vitro in a concentration-dependent manner via increased K^+^ efflux of the cells [114]. However, these effects were antagonized by 1–2% serum bovine albumin, and they demonstrated that gossypol is a specific and potent membrane active agent capable of injuring the cell membrane [114]. The administration of gossypol may be responsible for alterations in the hepatic metabolizing system [115], especially for the inhibition of microsomal enzymes [116]. Also, an increased heme degradation via stimulation of heme oxygenase activity in the liver and the kidney [117] contributes to the toxicity profile of gossypol [118]. In rats, glucuronidation was the only metabolic pathway for gossypol. However, the excretion of unmetabolized gossypol into bile was also noted as an important clearance mechanism [119]. Pharmacokinetic and toxicological studies of gossypol [11,120,121,122,123] indicate a species-specific differential sensitivity to the action of (±)-gossypol [112,122], and data regarding the pharmacokinetics and pharmacodynamics in humans is incomplete and heterogenic. Therefore, there is an urgent need to determine these for further investigations.

### 4.2. Potential Benefit and Current Limitations

AT-101-related strong antitumor effects and improved survival were recently shown in individuals with gastroesophageal carcinoma [95]. This is in line with data from in vitro [29] as well as from animal models [67,124,125,126]. Clinical trials, analyzed within this systemic review, demonstrated benefits for some patients, suggesting that further testing of AT-101 as an anti-tumor agent is advisable. However, there are also questions that have not yet been answered in these clinical trials, such as the determination of the AT-101 dosing levels, dosing frequency, and duration of treatment. Currently, there is no consensus on these parameters. Until now, gossypol/AT-101 has only been administered orally to cancer patients. AT-101 was determined to have the best therapeutic index compared to the other enantiomers and was suggested as a drug candidate for further clinical trials [89]. Based on Chinese contraceptive trials, a dose of 20 mg/day [103] was considered as the basic value. Neither testing AT-101 as a mono-therapy (20–70 mg/day) [90,91,93,94] nor in combination with chemotherapy (10–40 mg/day) [57,96] showed a dose related benefit in the participants, with the exception of Song et al. [95] (Table 1 and Table 2). Concerning dose frequency, two major regimes were evaluated: continuously or a cycle-defined administration. Continuous administration of AT-101 was applied by four investigators [54,89,93,94] (Table 1). Using a cycle-based regimen, AT-101 was given for 21 days of a 28-day cycle as monotherapy [90,91,92] or in combination with other standard chemotherapies (Table 2). In addition, Stein et al. examined AT-101 together with ADT on a daily basis [98]. An alternative cycle-based regimen was the administration of AT-101 on days one to three or one to five of a 21 days cycle concurrently in addition to the respective standard therapy for cancer patients. Among these, intravenously applied medication derived from natural plant products (paclitaxel with 150–175 mg/m^2^) [88], etoposide with 120 mg/m^2^ [99], or their synthetic derivatives docetaxel with 75 mg/m^2^ [97,101] and topotecan with 1.25 mg/m^2^ [102] were frequently used as a first combination partner, whereas 60–100 mg/m^2^ of cisplatin [57,96,99] or carboplatin (AUC 5/6 on day 1 of each cycle [88] were the second composite. Chemotherapy was commonly given intravenously at the beginning of the treatment cycle following the treatment- free period (19–16 days). Metronomic dosing was tested by Swiecicki et al. for the first time [97]. Thereby, the conductors of this trial compared three various dosing regimens: 75 mg/m^2^ docetaxel alone at the beginning of cycle, 75 mg/m^2^ docetaxel and 40 mg twice daily on days one to three as a “pulse dosing” and 75 mg/m^2^ docetaxel with 20 mg of AT-101 daily on days 1–14 of a 21 day cycle as a second metronomic regimen. The metronomic regimen was investigated because malignant cells may have varying rates of replication, and slow dividing cells may be less affected by high dose episodic chemotherapy, whereas the addition of a continuous agent may lead to tumoricidal synergistic effects [97]. In contrast to “pulse dosing” regimens, the combination containing low dosed compounds (AT-101, docetaxel, fluorouracil) and radiation showed a significant benefit in cancer patients [95]. Thereby, 10 and then 20 mg/day AT-101, docetaxel (20 mg/m^2^ as bolus) and fluorouracil (225–300 mg/m^2^) were given daily from Monday until Friday in addition to a radiation dose of 50.4 Gy (distributed over 28 fractions). However, it is important to mention that Song et al. examined this regimen in treatment naive patients [95] in contrast to the majority of AT-101 clinical trials, where heavily pre-treated and/or treatment resistant patients with different therapeutic backgrounds participated. Based on the available evidence, some recommendations can be made for future studies. These include that AT-101 dosing should be 10 mg/day at a minimum and not exceed 40 mg/day to avoid dose-limiting toxicities. Continuous administration of low dose AT-101 seems to be better tolerated. The combination with docetaxel and radiation for at least five cycles appears to be a promising approach [95,97].

### 4.3. Dose-Limiting Toxicities

Conductors of clinical trials that have examined the use of orally administered gossypol/AT-101 in cancer patient populations draw their attention to the fact that it is not yet clear why some patients respond to treatment and others do not. It is possible that the success of the therapy is limited by the occurrence of AT-101-related side effects and/or with SAEs. The incidence of these should be strictly monitored under AT-101 supplementation. Based on antifertility trials, AEs associated with gossypol at 60–70 mg/day include change in appetite, fatigue, dry mouth, diarrhea, and transaminase elevation [15]. At doses of 20 mg/day, the effects were less and included weakness, decrease or increase in appetite, dry mouth, and nausea [15]. Regarding reported dose-limiting toxicities in tumor patients, it is evident that these can be mainly categorized into hematologic, cardiac, dermatologic, gastrointestinal, hepatic, and metabolic events as well as nutritional behavior and general disorders (like fatigue, headache, and insomnia). The most common reported hematological toxicities were anemia, leukopenia, thrombocytopenia, and neutropenia [54,88,90,91,93,96,97,98,99,100,101,102]. Therefore, additional administration of (myeloid growth factors) filgrastim, pegfilgrastim, or erythropoietin was successfully applied [88,99,102]. Dermatologic DLTs were readily treated with topical steroids and diphenhydramine and resolved with drug discontinuations [54]. There are numerous trials describing the elevation of troponin levels or cardiac abnormalities [90,95,99,101]. Regarding gastrointestinal AEs noted in treated patients, nausea, vomiting, diarrhea, ileitis, abdominal pain, and constipation are the most common [54,88,90,91,92,94,95,98,102,107]. Generally, depending on the tested treatment regimen, 20–60 mg/day was suspected to cause elevation in liver parameters (AST/ALT, albumin, and bilirubin) and as DLTs [88,90,91,92,93,94,98,99,102]. In addition, electrolyte imbalances suchhypokalemia or hypercalcemia or other abnormalities of salt metabolism were also commonly reported [54,90,91,92,93,94,98,99]. In summary, dermatological and hematological AEs could be managed by additional administration of symptom-related drugs. Gastrointestinal disorders like emesis could be resolved with domperidone and prochlorperazine [89], and diarrhea with antidiarrheals [54,88,92,98,102]. Moreover, treatment of other AEs is manageable by dose reduction, which might be positively associated with a better adherence to therapy. However, whether dose reduction has a negative impact on treatment outcomes is not clear.

### 4.4. Association of Gossypol/AT-101 with Cancer Parameters

In addition, when considering patient response rates and/or life extension with respect to the regimen tested, the question arises as to what extent the administered gossypol/AT-101 dose correlates with the measured plasma levels or whether blood gossypol/AT-101 levels have an influence on the severity and/or course of the disease. In a cohort with advanced human cancer, serum gossypol levels were measured in seven patients approximately one day after treatment [89]. Gossypol was detectable in the serum, but no clear correlation was found between serum drug levels and the gossypol dose. The plasma gossypol levels achieved in the patients with metastatic adrenal cancer after gossypol supplementation ranged from 83 to 1142 ng/mL [94]. In general, gossypol levels in patients with tumor responses were indistinguishable from levels in patients without response. Notably, after discontinuation of gossypol the estimated half-life was noted to be 2.9 ± 0.9 weeks. Partial therapy responses were evident for doses of 0.6–0.8 mg/kg per day or 40–60 mg per day, respectively, and plasma levels ranged from 83–547 ng/mL. Therefore, no recommendation on the minimum effective blood gossypol concentration could be established. Higher doses of gossypol and higher plasma gossypol concentrations did not correlate with increased tumor response [94]. After testing gossypol in patients with recurrent adult malignant gliomas, plasma levels of gossypol were assessed four to eight weeks after starting therapy in 10 patients [93]. Thus, no difference between mean plasma levels in responders (115 ng/mL) and non-responders (129 ng/mL) was noted. Plasma gossypol levels, determined in a patient cohort of refractory metastatic breast cancer (165–465 ng/mL), were consistent with other clinical trials of gossypol [54]. These were analyzed after four weeks of treatment and did not correlate with the dose of gossypol (30–40 mg). In contrast to the above-described trials, based on the pharmacokinetic profiles established from patients with head and neck cancer, a dose-dependent increase in plasma concentration was shown, peaking between 1.5 and 2.5 h at approximately 300 and 700 ng/mL for the 10 mg and 20 mg dose levels, respectively [57]. Pharmacokinetic analysis was done after patients with solid tumors were treated with AT-101 in combination with paclitaxel and carboplatin [88]. It was shown that oral administration of AT-101 did not alter the pharmacokinetics of paclitaxel based on the collected parameters (t_1/2_, AUC, and clearance of AT-101), but was associated with inter-individual variability. A comparison was performed of the pharmacokinetics of AT-101 alone with those protocols in which AT-101 was given in combination with cisplatin and etoposide [99]. The maximum plasma concentration of gossypol was reached approximately three hours (range 2.86–3.94) after oral administration, indicating a slow absorption capacity. Administration of cisplatin and etoposide did not result in significant changes in AT-101 levels.

Treating patients with metastatic adrenal cancer revealed no definite effect of gossypol on hormone synthesis [94]. In men with castrate-resistant prostate cancer, evidence for single-agent drug activity was seen in three patients [92]. Thereby, PSA levels were reduced more than 50% compared to baseline in some patients and non-significant PSA level decreases were measured in many study participants. Stein et al. hypothesized that the addition of the Bcl-2 inhibitor AT-101 to ADT could contribute to increase the number of patients with undetectable PSA blood levels [98]. This study protocol (combined treatment of AT-101 and ADT) was tested in a cohort of patients with newly diagnosed castration sensitive metastatic prostate cancer. Study results from the Southwestern Oncology Group (SWOG) 9346 trial in 2006 showed that 48% of patients achieved undetectable PSA blood levels (≤0.2 ng/mL) at the end of seven months ADT [127]. However, the combination of AT-101 and ADT compared with ADT alone was not able to increase the percentage of patients attaining undetectable PSA. Here, only 31% of patients achieved undetectable PSA blood levels (≤0.2 ng/mL), and an additional 25% of patients normalized their PSA levels to equal or less than 4 ng/mL. Looking for predictors of sensitivity to ADT, chromodomain helicase DNA-binding protein (CHD1) was assessed in patient’s peripheral blood mononuclear cells. In a small number of patients, CHD1 correlated not significantly with therapeutic activity defined as high sensitivity for PSA values. In a large cohort of patients with metastatic castration-resistant prostate cancer, treatment with AT-101 in combination with docetaxel and prednisone as a first-line therapy lead to a significant PSA reduction of ≥30% in 66% compared to 54% of controls [100].

Correlative studies were performed to evaluate special tumor markers for determination of AT-101 treatment success. Targeting VEGF-BCL2-CXCL8 (chemokine C-X-C motif Ligand 8 (CXCL8 or Interleukin-8 (IL-8)) pathway via AT-101 and docetaxel showed minor but not significant differences between treatment arms with patients with locally advanced or metastatic head and neck cancer [97]. Analysis of peripheral blood mononuclear cells (PBMCs) from patients with chemotherapy-sensitive relapsed extensive-stage small cell lung cancer revealed marked variability in caspase activation between patients but no consistent evidence of apoptosis induction by AT-101 [91]. Also, no statistically significant decreases of Bcl-2 and Caspase 3 protein levels or increased apoptotic activities were detected in subjects with solid tumors after a triple therapy of AT-101, paclitaxel, and carboplatin [88]. In contrast, gossypol plasma levels were related to retinoblastoma (Rb) protein and Cyclin D1 expression in serial biopsies of refractory metastatic breast cancer [54]. In three of four tumor specimens, an increase in Rb protein and a decrease in cyclin D1 were noted by immunohistochemical analysis. Moreover, a decrease of >50% regarding tumor markers like serum breast cancer antigen BR2729 (BR2729 or CA15-3) and/or carcinoembryonic antigen (CEA) was experienced in some patients. In future studies, determination of a blood- or tissue-derived marker associated with AT-101 treatment could be an important contributor to the success of AT-101 therapy and monitoring.

### 4.5. Future Perspectives

In summary, results from the analyzed studies are heterogeneous regarding the correlation between dosing, plasma levels, tumor markers, and outcome. Therefore, these have only been investigated in small cohorts and not in the whole study group. In addition, there is a lack of comparability of the studies, as in some cohorts patients with different tumor entities (mixed population) are included [88,89,99]. Further studies are needed to elucidate the exact mechanisms of absorption, interactions with enzymes involved in drug metabolism, and degradation as well as the extent and pathways of excretion of AT-101. Furthermore, it must be considered that gossypol/AT-101 was administered to patient cohorts with different therapy experience (naive, heavily pre-treated or resistant). The sensitivity of different tumor entities to certain standard chemotherapeutics could also influence the success of the therapy. As investigated by Wang et al., patients with high expression of apurinic/apyrimidinic endonuclease 1 (APE1) and therefore to cisplatin were only included for examination of the AT-101 and cisplatin regimen in a cohort of advanced non-small cell lung cancer [96]. In addition to these clinical studies, laboratory research has attempted to elucidate the synergetic mechanism of action of AT-101 and other agents in cancer cells. In non-small cell lung cancer, AT-101 selectively inhibited cell proliferation and induced apoptosis via targeting EGF receptor with L858R/T790M mutations [128], overcame EGFR tyrosine kinase inhibitor resistance [129], and enhanced gefitinib sensitivity in cancer cells with EGFR T790M mutations [67]. However, the association between the therapeutic effect of gossypol and genetic alterations needs to be clinically investigated for other tumor entities or subtypes of cancer cells that may be affected by AT-101 treatment. Therefore, before well-defined clinical recommendations can be made, further research is required to establish the therapeutically effective dose of AT-101, the best combination with chemotherapeutic agents, and the therapeutic window.

## 5. Conclusions

In clinical trials, evidence supporting the anticancer effects of gossypol/AT-101 as a single agent or in combination with standard therapy is mixed. Solid tumors were evaluated, but very little is known regarding hematological malignancies. In trials that used oral AT-101 in combination with concurrent standard therapies, it is unclear which agent delivered which effects. A recent study combined AT-101 with two chemotherapeutic agents and radiation as metronomic therapy, and achieved significant benefits. Such trials could further elucidate the synergistic anticancer effects of AT-101 when used in combination with anti-neoplastic agents. The current research indicates that AT-101 is well tolerated at low doses and could have an impact on tumor markers. Only a few randomized clinical trials have been performed, showing a trend toward increased OS and PFS. More high-quality placebo-controlled trials are needed to strengthen the present evidence to support AT-101 as a treatment option. Until these trials are completed, patients could be informed of the investigational status of AT-101 as a potential cancer treatment with meaningful risk for AEs due to its high toxicity capabilities.

## Figures and Tables

**Table 1:** Oral application of single-agent gossypol/AT-101 investigated in cancer patients in Phase I and II trials.

NCT Number Publication Date Country Reference	Tumor Entity Patient Diagnosis n	Trial Design	Treatment Type and Frequency	Toxicity	Reported Outcomes/Conclusions
NCT00848016 2019 USA [90]	patients with histologically confirmed metastatic, recurrent, or primarily unresectable advanced adrenal cortical carcinoma n = 29	nonrandomized, single-center phase II	20 mg AT-101 orally daily for 21 days of 28-day cycles patients pre-treated a total of 80 cycles	AEs grade ≥4: cardiac troponin elevations and hypokalemia AEs grade 3: GI disorders, hypokalemia, AST/ALT elevation, fatigue	29 of a targeted 44 patients accrued 27/29 patients had incurred PD PR: no patient SD: eight patients for median duration of 3.8 (1.8 to 10.1) months median time of progression 1.9 months, mOS: 8.5 months was closed at the futility interim analysis due to lack of activity
NCT00773955 2011 USA [91]	recurrent chemosensitive ES-SCLC n = 14	phase II	20 mg AT-101 orally daily for 21 days of 28-day cycle up to six cycles	grade 3/4 toxicities AE grade 3/4 in four patients, nausea, vomiting, fatigue, anorexia AEs grade 3, hematologic, in two patients no grade 4 toxicities	OR: no patients SD: three patients median time of progression 1.7 months mOS: 8.5 months terminated due to failure to pass the pre-specified interim analysis per study design
NCT00286806 2009 USA [92]	progressive CRPC n = 23	open-label, multicenter, phase I/II	30 mg AT-101 as starting dose (reduced later to 20 mg) for 21 days of 28-days cycle chemotherapy naive patients ≥eight weeks of therapy	most frequent observed AE (any grade) of GI origin AE grade 4 elevation of AST/ALT due to the high incidence of grade 3 small intestinal obstruction a reduction to 20 mg/day for all patients	decline in PSA over 50% in two patients no OR, SD for 24 weeks in two patients AT-101 administered at 20 mg/day for 21 of 28 days is well-tolerated modest single-agent activity of AT-101 phase I was terminated earlier due to emerging data from other trial
NCT n. a. 2001 USA [54]	refractory metastatic breast cancer n = 20	phase I/II	30–50 mg AT-101 daily patients were pre-treated with doxorubicin and paclitaxel for advanced disease	grade 1/2 toxicities: nausea, fatigue, emesis, dysgeusia and diarrhea DLT dermatologic (grade 3) for 50 mg/day no grade 4 toxicities occurred	blood gossypol levels are 10-fold lower than in vitro levels no clear correlation between plasma drug levels and the gossypol dose MR: 1 and SD: two patients no partial or CR no therapeutic responses observed
NCT n. a. 1999 USA [93]	pathologically confirmed glial tumors which had recurred after radiation therapy n = 27	phase II	10 mg racemic gossypol acetic acid orally BID daily all patients had previous irradiation different co-medication permitted	mild toxicity thrombocytopenia two patients hypokalemia 5 patients grade 2 hepatic toxicity and peripheral edema three patients	PR: two patients (for eight and 78 weeks) SD: four patients for at least eight weeks PD: 21 patients no difference plasma levels in responders and non-responders study stopped based on low response rate in poor-prognosis, unselected group of patients
NCT n. a. 1993 USA [94]	metastatic adrenal cancer n = 21	phase I	30–70 mg racemic oral gossypol daily (increasing by 10 mg/day every 2 days) mitotane and suramine as prior treatment	gossypol generally well tolerated 1 SAE: abdominal ileus AEs: dermatologic, transient transaminitis, GI disorders, hypokalemia	18 patients hat at least 18 weeks gossypol treatment PR: three patients (≥50% decrease in tumor volume) MR: one patient PD: 13 patients oral gossypol can be used relatively safely administrated responses seen in patients who had failed other chemotherapeutic regimens no significant decrease in steroid secretion
NCT n. a. 1992 UK [89]	advanced human cancer n = 34	phase I	racemic gossypol acetic acid as dose escalating regimen part I: weekly escalating doses of gossypol ranging from 30 to 180 mg part II: repeat doses (30 mg), which were given initially twice weekly, then daily and, finally, twice daily	no major adverse events no evidence of hematological or biochemical disturbance daily median limiting dose = 30 mg, weekly = 120 mg toxic side effects, emesis is dose related (severe in 13/16 patients), diarrhea, lethargy no evidence in liver metastases, bone marrow toxicity, hypokalaemia related to gossypol	no clear correlation between serum drug levels and gossypol dose 23 patients completed at least three weeks treatment 20 patients assessable for response no tumor regression SD: three patients (for 16, 23 and 19 weeks), PD: 20 patients achieved gossypol blood levels were lower than in vitro (−)-enantiomer/AT-101 suggested to use in further clinical trials

Abbreviations: ADT, androgen deprivation therapy; AE, adverse event; ALT, alanine aminotransferase; AST, aspartate aminotransferase; BID, twice daily; CR, complete response; CRPC, castrate-resistant prostate cancer; DLT, dose-limiting toxicity; ES-SCLC, extensive stage—small cell lung cancer; GI, gastrointestinal; mOS, median overall survival; MR, minor response; n, number of subjects; n. a., not available; OR, objective response; OS, overall survival; PD, progressive disease; PFS, progression-free survival; PR, partial response; PSA, prostate-specific antigen; SAE, serious adverse event; SD, stable disease.

**Table 2:** Application of gossypol/AT-101 in combination with standard chemo- and radiation therapies in Phase I and II trials.

NCT Number Publication Date Country Reference	Tumor Entity Patient Diagnosis Number (n)	Trial Design	Treatment Type and Frequency	Concurrent Treatment	Toxicity	Reported Outcomes/Conclusions
NCT00561197 2021 USA [95]	GEC n = 13	open label, phase I/II	10 (starting dose) or 20 mg (final dose) AT-101 taken orally Monday through Friday of each week of chemoradiation	docetaxel (20 mg/m^2^ as bolus once a week × 5), IV fluorouracil (225–300 mg/m^2^ as low-dose continuous infusion daily from Monday through Friday × 5), IV radiation (50.4 Gy in 28 fractions)	most common AE are GI tract related a total of 9 SAE irrespective of relationship to AT-101 troponin I levels were elevated in four patients, AT-101 related no ECG abnormalities or cardiac symptoms no AEs required dose reduction DLT	cCR: 11/13 patients median duration of cCR: 12 months (3–59 months) PFS: 52 weeks with recurrences in 10 of 13 patients salvage surgery could be performed in only four patients five of 13 patients had expired mOS was not reached at a median follow-up time of two years none of the clinical variables correlated with OS or PFS survival much longer than expected phase 2 cloud not be completed, study stopped due to sponsor decision
NCT01977209 2020 China [96]	advanced NSCLC n = 62 (co = 31, eg = 31)	double-blind, randomized, placebo-controlled, phase III	eg = 20 mg gossypol once daily on days 1–14 of 21-day cycle as gossypol acetate tablets (20 mg/tablet)	eg = docetaxel (75 mg/m^2^) cisplatin (75 mg/m^2^) co = docetaxel (75 mg/m^2^) cisplatin (75 mg/m^2^) IV, both on day 1 of 21-day cycle	no significant differences in RR and SAE between the groups no treatment-related deaths or discontinuation of treatment due to toxicity no significant increase in toxicity in eg vs. co grade 3 toxicity: anemia grade 1 or 2 toxicity: neutropenia, asthenia, fatigue, dyspnea, anemia, leukopenia, thrombocytopenia, headache	no significant differences in PFS and OS between eg and co PD: 17 vs. 21 patients mPFS: 7.43 vs. 4.9 months mOS: 18.37 vs. 14.7 months six-month PFS rate: 45.2% vs. 22.6% 12 months survival achieved: 17 vs. 10
NCT00891072 2020 USA [88]	advanced solid tumors n = 24	open label, dose escalating, nonrandomized, single-center, phase I	40 mg AT-101 orally every 12 h on days one, two and three of each 21-day cycle	varying dose levels of paclitaxel (150 or 175 mg/m^2^, 1 h after AT-101) carboplatin (AUC 5 or 6, after paclitaxel) both IV on day 1 of each 21 days cycle planed for a maximum of eight cycles in absence of PD	DLTs as abdominal pain and ALT increase (n = 2) significant GI toxicities most common fatigue, nausea, metabolism, nutrition disorders, and anorexia moderate hematologic toxicity (anemia, thrombocytopenia, neutropenia, and leukopenia)	evidence of efficacy in five subjects (1 CR, 4 PRs) 12 patients with SD for 4–12 cycles combination of AT-101 paclitaxel and carboplatin was safe and tolerable based on the modest clinical efficacy seen in this trial, this combination will not be further investigated
NCT01285635 2016 USA [97]	un-resectable, recurrent, or locally advanced or metastatic HNSCC, not amenable to curative radiation or surgery n = 35	open label, randomized, phase II	pulse dose: 40 mg AT-101 BID on days one to three of 21-day cycle metronomic dose: 20 mg AT-101 daily on days 1–14 of 21-day cycle	docetaxel (75 mg/m^2^) on day one of 21-day cycle, IV planed 10 cycles	two patients discontinued treatment due to toxicity 12 patients had dose modifications due to hematologic toxicities hematologic toxicities are common treatment related toxicities 11 episodes of grade 3–4 lymphopenia five episodes of grade 3–4 anemia	combined therapy with AT-101 and docetaxel does not provide an incremental clinical benefit in R/M HNSCC AT-101 containing regimens was well tolerated 74% had a clinical benefit (CR, PR, or SD) 11% RR 66% achieved SD mPFS was 4.3 months (0.7–13.7) mOS of 5.5 months (0.4–24) the six-month PFS was 24% on interim analysis after enrollment of 35 patients a lack of improvement in survival was noted hence the trial was stopped due to futility
NCT00666666 2016 USA [98]	newly diagnosed castration-sensitive metastatic prostate cancer n = 55	open label, multicenter study, phase II	20 mg AT-101 orally daily for 21 days of a 28-day cycle, up to 8 cycles	ADT with a luteinizing hormone-releasing hormone agonist (bicalutamide), started 6 weeks before initiation and delivered using physician’s choice planed up to eight cycles	treatment discontinuation in 35% (19/55) of patients due to AEs SAE in 12 patients SAEs in seven patients related to study therapy the majority of related AEs were GI and nervous system disorders, increased AST/ALT, of grade 1/2 toxicities	data analysis at the 7.5 months’ time point from initiation of ADT demonstrated 17 (31%) achieved an undetectable PSA (≤0.2 ng/mL) 14 (25%) had PSA > 0.2 and ≤4.0 ng/mL two (4%) had PSA > 4 ng/mL no additional patients developed undetectable PSA after 7.5 months of ADT combination of ADT and AT-101 did not meet the prespecified level of activity for further development of this combination
NCT n. a. 2015 Netherlands [57]	locally advanced inoperable head and neck cancer (HNSCC) n = 14	phase I/II	dose-escalating oral administration 10 mg (starting dose, n = 13) and 20 mg (n = 1) AT-101 daily in a two-weeks daily schedule every three weeks	cisplatin (100 mg/m^2^) 3 × weekly, IV chemoradiotherapy (70 Gy delivered in 35 fractions over 7 weeks)	not described, only pharmacokinetic table available	pharmacokinetic analysis of patient blood samples taken between 30 min and 24 h after intake of AT-101 showed a dose-dependent increase in plasma concentration with peak levels up to 300–700 ng/mL between 1.5 and 2.5 h after intake at daily doses of 10–20 mg, plasma levels peaked around 2 h after intake, suggesting slow absorption maximum plasma concentrations were in the micromolar range, corresponding to those that induced radiosensitization in vitro
NCT00544596 2014 USA [99]	patients with advanced solid tumors (1. cohort), and an expanded cohort of patients with ES-SCLC (2. cohort, n = 7) n = 27	open label, dose escalating, phase I	20–40 mg AT-101 orally BID on days 1–3 of a 21-day cycle	cisplatin (60 mg/m^2^) on day one etoposide (100 mg–120 mg/m^2^) on day 1–3 both IV, 21-day cycle	no evidence of cumulative toxicity high incidence of grade 3–4 neutropenia and leukopenia improvement after inclusion of filgastrim nine patients (33%) hat SAEs considerable rate of GI toxicities least grade 1–2 grade 3/4 treatment-related toxicities included: diarrhea, increased AST, neutropenia, hypophosphatemia, hyponatremia, myocardial infarction and pulmonary embolism	18/20 patients assessable for response in a first cohort four patients with PR 10 patients with SD four patients with PD 6/7 18/20 patients assessable for response in a first cohort five patients with PR AT-101 with cisplatin and etoposide is well tolerated with filgastrim support
NCT00571675/ NCT00286793 2012 USA/Russian Federation [100]	metastatic CRPC n = 220	double-blind, placebo-controlled, two-arm trial with 1:1 randomization of phase II	40 mg AT-101 BID on days 1–3 of 21-day cycle or placebo (co)	docetaxel (75 mg/m^2^), IV on day one of 21-day cycle prednisone 5 mg orally BID median number of cycles = 8/9	higher incidence of grade 3/4 AEs in the e.g., including cardiac events, lymphopenia, neutropenia, pulmonary embolism and peripheral neuropathy	mOS: 18.1 vs. 17.8 months (eg vs. co) mPFS: 11.0 vs. 10.3 months potential benefit was observed in high-risk patients with OS of 19 vs. 14 months PSA reductions of ≥30% were seen in 66% vs. 54% of patients PSA reductions of ≥50% in 54% vs. 46% of patients measurable disease control rates 93% vs. 80%
NCT00544960 2011 USA/Russian Federation/Ukraine [101]	advanced or metastatic NSCLC n = 105	double-blind, randomized (1:1), placebo-controlled phase II	40 mg AT-101 BID on days 1–3 of 21-day cycle (dose reduction because of possible toxicity to 30 and 20 mg BID) or placebo	docetaxel (75 mg/m^2^) on day 1 of 21-day cycle (dose reduction steps because of possible toxicity by 15 mg/m^2^ each) maximum of 10 cycles were allowed	AE: fatigue, anemia, dyspnea, headache (grade 1/2) no cases of small bowel obstruction no statistically significant differences in SAE between AT-101 and placebo AT-101 AE profile indistinguishable from the base docetaxel regimen	docetaxel plus AT-101 vs. docetaxel plus placebo (eg vs. co) PFS: 7.5 vs. 7.1 months OS: 7.8 vs. 5.9 months AT-101 plus docetaxel was well tolerated
NCT00397293 2010 USA [102]	relapsed and refractory SCLC, who had progressed on prior platinum-containing chemotherapy n = 36	open-labeled, multicenter, phase I/II	40 mg AT-101 daily on days 1–5 of a 21-day cycle	topotecan (1.25 mg/m^2^), IV on days 1–5 of 21-day cycle	DLT in at 40 mg AT-101 DLT non-hematological not noted AEs in at least 10% most common were hematologic and GI toxicities (grades 1 and 2)	in the sensitive relapsed cohort (n = 18): CR = 0, PR = 3, SD = 10, PD = 4 in the refractory cohort (n = 12): CR/PR = 0, SD = 5, PD = 5 due to failure of pre-specified endpoints no second stage of the phase II study median time to progression in the sensitive-relapsed cohort was 17.4 vs.11.7 weeks in the refractory cohort 40 mg/d AT-101 can be safely combined with topotecan (1.25 mg/m^2^)

Abbreviations: ADT, androgen deprivation therapy; AE, adverse event; ALT, alanine aminotransferase; AUC, area under the concentration time curve; AST, aspartate aminotransferase; BID, latin: bis in die (twice a day); co, control; cCR, clinical complete response; CR, complete response; CRPC, castrate-resistant prostate cancer; DLT, dose-limiting toxicity; ECG, electrocardiogram; eg, experimental group; ES-SCLC, extensive-stage small cell lung cancer; GEC, gastroesophageal carcinoma; GI, gastrointestinal; HNSCC, head and neck squamous cell carcinoma; IV, intravenously; mOS, median overall survival; mPFS, median progression-free survival; n, number of subjects; n. a., not available; NSCLC, non-small cell lung cancer; OS, overall survival; PD, progressive disease; PFS, progression-free survival; PR, partial response; R/M, recurrent/metastatic; RR, response rate; SAE, serious adverse event; SCLC, small cell lung cancer; SD, stable disease.

## Data Availability

No new data were created or analyzed in this study. Data sharing is not applicable to this article.

## Abbreviations

ADT	androgen deprivation therapy
AE	adverse events
AIF	apoptosis-inducing factor
ALT	alanine aminotransferase
APE1/Ref-1	apurinic/apyrimidinic endonuclease 1/redox enhancing factor-1
AST	aspartate aminotransferase
AUC	area under the concentration time curve
BID	latin: bis in die; twice a day
Bcl-2	B-cell lymphoma 2
BH3	Bcl-2 homology domain 3
CEA	carcinoembryonic antigen
CHD1	chromodomain helicase DNA-binding protein
co	control
cCR	clinical complete response
CR	complete response
CRPC	castrate-resistant prostate cancer
CTCAE	Common Terminology Criteria for Adverse Events
CXCL8	chemokine C-X-C motif ligand 8
DCR	disease control rate
DLT	dose-limiting toxicity
DNA	deoxyribonucleic acid
ECG	electrocardiogram
ECOG	Eastern Cooperative Oncology Group
eg	experimental group
EGFR	epidermal growth factor receptor
ES-SCLC	extensive stage—small cell lung cancer
GEC	gastroesophageal carcinoma
GI	gastrointestinal
HDAC	histone deacetylase
HDACi	histone deacetylase inhibitor
HNSCC	head and neck squamous cell carcinoma
IL-8	interleukin-8
IV	intravenously
LHRH	luteinizing hormone-releasing hormone
MOMP	mitochondrial outer membrane permeabilization
mOS	median overall survival
mPFS	median progression-free survival
MR	minor response
MTD	maximally tolerated dose
n. a.	not available
ORR	objective response rate
OR	objective response
OS	overall survival
P-gp	p-glycoprotein
PBMC	peripheral blood mononuclear cell
PCWG	Prostate Cancer Clinical Trials Working Group
PD	progressive disease
PFS	progression-free survival
PO BID	latin: per os bis in die; orally, twice a day
PO QD	latin: per os quaque die; orally, once a day
PR	partial response
PSA	prostate-specific antigen
R/M	recurrent/metastatic
Rb	retinoblastoma protein
RECIST	response evaluation criteria in solid tumors
ROS	reactive oxygen species
RR	response rate
SAE	serious adverse event
SCLC	small cell lung cancer
SD	stable disease
SOX9	SRY-box transcription factor 9
SWOG	Southwestern Oncology Group
VEGF	vascular endothelial growth factor
YAP1	yes-associated protein 1
